# An Overview of Network-Based and -Free Approaches for Stochastic Simulation of Biochemical Systems

**DOI:** 10.3390/computation6010009

**Published:** 2018-01-31

**Authors:** Abhishekh Gupta, Pedro Mendes

**Affiliations:** Center for Quantitative Medicine and Department of Cell Biology, University of Connecticut School of Medicine, 263 Farmington Av., Farmington, CT 06030-6033, USA

**Keywords:** stochastic simulation, modeling, network-based, network-free, rule-based modeling, systems biology

## Abstract

Stochastic simulation has been widely used to model the dynamics of biochemical reaction networks. Several algorithms have been proposed that are exact solutions of the chemical master equation, following the work of Gillespie. These stochastic simulation approaches can be broadly classified into two categories: network-based and -free simulation. The network-based approach requires that the full network of reactions be established at the start, while the network-free approach is based on reaction rules that encode classes of reactions, and by applying rule transformations, it generates reaction events as they are needed without ever having to derive the entire network. In this study, we compare the efficiency and limitations of several available implementations of these two approaches. The results allow for an informed selection of the implementation and methodology for specific biochemical modeling applications.

## 1. Introduction

Research in systems biology has been increasingly supported by computational models of biochemical reaction networks. These models are studied either through a deterministic approach, using differential equations to represent the temporal changes of the concentrations of the chemical species, or via a stochastic approach based on the chemical master equation (CME) and solved through Monte Carlo simulation algorithms. Although the deterministic approach of solving a set of differential equations by numerical integration is fast and widely adopted, it is unable to estimate the variance of the species concentrations and can become inaccurate for systems with a small number of particles [1]. In these situations, the stochastic approach of using a Monte Carlo simulation algorithm for evaluation of the CME is preferred.

The CME is a very high dimension differential equation that describes the evolution of the entire state space. Direct solutions of the CME are rare and apply only to very small systems. In practice, the CME is solved by applying a simulation algorithm that provides an exact solution in the Monte Carlo sense (i.e., by summation of many simulated trajectories). Gillespie derived an algorithm that satisfies this requirement [2]. This is often referred to as the Gillespie algorithm, although Gillespie himself referred to it as the *stochastic simulation algorithm* (SSA). In fact, Gillespie provided two variants of the algorithm, the *direct method* and the *first reaction method*, with the direct method being the most widely used. Gillespie’s SSA has been further improved for computational efficiency [3–5] and there have been several implementations of these algorithms in software for computational systems biology. For example, there are software applications, such as COPASI [6], VCell [7], and StochPy [8], which provide user-friendly platforms to create and simulate models using the SSA and include other features to further analyze the model and simulation results. Furthermore, there are other lightweight programs developed specifically to simulate models using the SSA, namely Dizzy [9], Gillespie2 [10], SGNS2 [11], RoadRunner [12] and pSSAlib [13]. All these simulators require the full set of reactions—the reaction network—to be enumerated beforehand and thus are sometimes termed “network-based”.

There are several cases in which a biochemical network is very large or limitless. A particularly common occurrence is given by some signal transduction networks that contain proteins with multi-site phosphorylation, leading to combinatorial numbers of chemical species and reactions between them [14–16]. Another case is that of the formation of polymers with an unlimited number of monomers. In order to model such systems, an approach has been developed in which sets of similar reactions are defined by rules that apply to sets of species specified by patterns [17–19]. This formalism results in a concise model specification of the underlying chemical kinetics [20,21]. The most common rule-based modeling languages are the BioNetGen language (BNGL) [17] and Kappa [22]. The BNGL simulator, BioNetGen [17,23], operates by deriving the reaction network specified in the reaction rules and then applying the SSA for simulation. On the other hand, rule-based simulators such as KaSim, PySB [24], RuleMonkey [25] and NFsim [19] carry out simulations directly on the basis of reaction rules without deriving the entire reaction network, and accordingly these have been termed “network-free”. At its core, all these simulators are based on Gillespie’s method, as rules are sampled at each time interval using a method equivalent to how reactions are sampled in the SSA.

In this review, we compare these two stochastic simulation approaches and several popular software implementations in the context of models with different complexity. The comparison addresses issues such as the number of particles, species, and reactions, as well as the length of the simulation.

## 2. Network-Based Approach

The stochastic formulation of chemical kinetics describes the time evolution of a well-stirred set of chemically interacting particles in thermal equilibrium within a fixed reaction volume [1]. The time evolution of the number of particles of each species in the volume, on the basis of the probabilities of all reactions that can occur in the system, is driven by the CME. As already mentioned, the CME is rarely solved analytically, mostly because the number of its terms grows exponentially with the number of species in the system.

An alternative to the analytical solution of the CME is to simulate the trajectories of molecular populations in exact accordance with the CME, as proposed by Gillespie [2] (the SSA). Each trajectory corresponding to a single SSA run represents an exact sample from the distribution defined by the CME. The steps of SSA can be summarized as follows:

1. **Initialize**: Set the time *t* = 0 and set up the initial state vector, propensities, and random number generators.
2. **Execute**: Using a suitable sampling procedure, generate random numbers and, on the basis of these, determine the next reaction to occur and the time interval.
3. **Update**: Update the molecule count, and if needed, recalculate the propensities. Output the system state.
4. **Iterate**: If simulation end time is not reached, go to step 2.

The two original, and statistically equivalent, sampling procedures for step 2 of the SSA are the direct method (DM) and the first reaction method (FRM) [2]. The DM samples two random numbers from the uniform distribution in the unit interval, and the time of next reaction (*τ*) is first generated according to the probability function of reactions. Using *τ*, the DM then generates the indices of reactions and selects the one to occur next. The FRM, using a random number, generates “tentative reaction times” (*τ_v_*) for all the reactions and then selects the reaction with the smallest *τ_v_*. Because the FRM needs to generate many more random numbers per iteration than the DM (for systems with three or more species), the DM is generally the procedure implemented for the sampling in step 2 of the SSA [2].

Gibson and Bruck proposed the next reaction method (NRM) [4] that can reduce the computational costs of the SSA significantly. In addition to using one random number per iteration, to reduce the time to update propensities and to find the smallest *τ_v_* value, the NRM uses an indexed priority queue to store the *τ_v_* values generated in previous iterations and to extract them whenever required. This results in a significant improvement in the runtime performance when compared to the FRM. This algorithm is exact as well as efficient. For large reaction networks and loosely coupled reaction systems, the NRM is significantly faster than both the FRM and the DM. This advantage, however, may not be significant for small systems, as the computational cost of maintaining the additional data structures required dominates the simulation time [5].

Other variants to accelerate the search for the next reaction in the SSA have been proposed, such as the optimized direct method (ODM) [5], the sorting direct method (SDM) [26], the partial-propensity direct method (PDM) [27], and the SSA with composition rejection algorithm (SSA–CR) [28–30].

Besides the exact algorithms mentioned above, many others have been proposed that can accelerate the simulation even further, but they do this by adopting approximations and no longer provide exact solutions. A popular method is the *τ*-leaping algorithm [31], which does not simulate each reaction event individually but rather steps a time-span *τ* and estimates how many and which reactions have happened meanwhile. Many other variants of this and other approximations have been proposed, including hybrid methods that partition the system into a part that is simulated using differential equations and another that uses the SSA or one of its variants (see review by Pahle [32]).

Most of the stochastic simulators provide options to choose between the DM and the NRM, for example, COPASI [6], StochPy [8], and Dizzy [9]. Other simulators use only one of these, with Gillespie2 [10] and RoadRunner [12] using only the DM, and SGNS2 [11] using only the NRM. The pSSAlib software [13] allows selection between the DM, the PDM, a sorting variant of the PDM (SPDM), and the SSA–CR. StochKit2 [33] provides several of these, including the SSA–CR, but automatically selects which algorithm to use.

## 3. Network-Free Approach

To address the combinatorial complexity in biological signaling networks [14], originating from multiple post-translational modifications and conformational changes, rule-based modeling approaches have been developed [15,17,18,20–22,34]. At the core of these approaches are *reaction rules* that represent groups of reactions. These rules refer to specific binding sites with or without specific ligands. Rules can also specify different states of a molecule (such as oxidized or reduced, phosphorylated or unphosphorylated, etc.). With rule-based modeling it is easy to specify, with a few rules, a complex set of combinatorial interactions in which several subunits can assemble into larger complexes and allow for modification of specific moieties. This type of model specification is therefore very useful for signal transduction networks in which these types of interactions are abundant.

The BNGL [17,35–37], the *κ*-language [18], and *ρ_bio_*-calculus [38] are some examples of formalisms developed for biochemical rule-based modeling. While the BNGL can be processed by different software applications (BioNetGen [23,36], DYNSTOC [25], RuleMonkey [39], and NFsim [19]), the other languages are mostly restricted to being processed by a single software package. The BioNetGen software package expands a BNGL rule-based model to a reaction network, which is then simulated using a variety of deterministic and stochastic network-based methods. However, when a rule-based model can result in a large reaction network, the expansion as well as the simulation of such a network becomes computationally expensive. For such scenarios, the generation of the reaction network can be avoided by a network-free simulation approach.

DYNSTOC [25] uses an agent-based null-event stochastic simulation approach based on an earlier package, STOCHSIM [40]. In this approach, each of the reactive molecular components are represented as a software object (agent), and these are tracked individually during the simulation. More specifically, for each fixed time increment, on the basis of a decision to select either one or two molecules for the next reaction, the reactants are first chosen randomly. Then the rules that qualify for the interaction on the basis of the chosen reactants are shortlisted, and for the reaction with the highest probability, an update is performed using a graph-rewriting operation.

Unlike DYNSTOC, which uses a fixed time-step, RuleMonkey [39] has a variable time increment, and rules are represented as pattern graphs. The simulation procedure is similar to the SSA [2], as the time increment and rule selection are based on the DM. Once a rule to execute next is chosen, the most potential reactants are selected on the basis of the pattern graphs and are then used to update the state of system. As such, RuleMonkey uses iterative updates to track rule rates exactly, avoiding null events that do not change the state of the system being simulated [41]. NFsim [19] is another rule-based simulator, using a generalized algorithm [42] also based on the SSA. Contrary to RuleMonkey, NFsim introduces null events in its implementation [41]. While both RuleMonkey and NFsim have been shown to perform similarly over a wide parameter range [41], NFsim has the additional capabilities of defining functional rates and coarse-grained rules. It uses an efficient representation of molecules, complexes, and rules as well as an optimized handling of reactant selection and transformation.

These network-free simulators, unlike the network-based SSA simulators, scale with the number of rules rather than the number of reactions and thus should be very efficient for systems in which a few rules can represent a large number of reactions [37]. While this is true for networks with limited interacting particles, the network-free simulators might not be as efficient, given they represent each particle individually. The particle-specific events, such as aggregation and polymerization, make the computational cost even higher. On the other hand, although the network-based simulators are dependent on the number of reactions, their efficiency is not affected greatly by the number of molecules [37]. As such, network-based simulators may be preferred for systems with large particle numbers and a moderate reaction network.

All of the approaches described have difficulties when there are large numbers of particles and a large reaction network. To address this situation, there have been efforts to develop hybrid methods [37,43–45]. The hybrid particle-population-based approach [37] is reported to be exact and efficient but requires a predefined partition of the system into network-free and -based parts. Because these hybrid approaches are based on the partitioning of the models, it is important to identify limits of both network-based and -free approaches such that automatic identification of different parts of the network could be created on the basis of this information [37].

## 4. Benchmarking Stochastic Simulation

### 4.1. Simulators

A survey of the literature reveals that several of the stochastic simulators described above are regularly used in computational systems biology but to our knowledge have never been compared for performance in a systematic way. Given that in stochastic simulation one must define the time-dependent distribution (or at least some statistics of this distribution, such as the mean and standard deviation), this usually requires repeating simulations many times; thus the performance of the simulators used may be a critical factor. We profiled a number of the most widely used simulators with a set of models of increasing complexity. The intention was not only to compare simulators on the basis of similar algorithms, but also to compare the different algorithms used. We note that we only considered software implementing methods that are exact solutions of the CME; approximate and hybrid methods were excluded.

We identified a series of commonly used and freely available network-based and -free simulators, which are listed in Table 1. Because we needed to profile these on the same computer to be able to compare them, only software packages that could be run on a local machine were included. Moreover, to be able to specify the same model across all of these, we used models specified with the BNGL; thus we restricted the selection to packages that could either process BNGL directly or could import models in the systems biology markup language (SBML) [46] (BioNetGen was used to generate the reaction network and export it in SBML format). Three exceptions were included: KaSim, which uses the *κ*-language rather than BNGL; pSSAlib, which has an implementation of the PDM and the SSA–CR; and StochKit2, which has an implementation of the SSA–CR. For KaSim, we translated the models into the *κ*-language. For pSSAlib, we created a program that converts the standard SBML into the specific dialect it can understand (while pSSAlib claims to read SBML, it requires specific annotations in the files). For StochKit, we used the SBML converter that was provided with that package.

### 4.2. Models

To compare the performance of simulators under different conditions, we selected models with increasing complexity, as quantified by the number of species and reactions. Table 2 summarizes the models considered in this study. The first two models, “multi-state” and “multi-site”, are conceptual and have been used for the illustration of basic biochemical networks. We expected the derivation as well as simulations of these models to be fast, as they have a small number of species and reactions. The remaining three models were originally formulated to study specific signaling networks. They are more complex than the previous two and allowed us to test the simulators under more realistic conditions.

The multi-state model is composed of three species, R, L, and A; R and L can form a complex “R.L”, and the latter can dissociate back to the monomers. The species A can bind to R, and it can exist in a phosphorylated or unphosphorylated state (see Figure A1 for details). This model was previously described in [17,25].

The multi-site model contains the same three species, but here A has three different phosphorylation sites; L binds A to any of its phosphorylated sites, and R binds A to its unphosphorylated sites (see Appendix A, Figure A2).

The Epidermal growth factor receptor (EGFR) signaling model describes the early signaling events in the epidermal growth factor receptor cascade [47]. Besides the epidermal growth factor and its receptor, the model consists of the adapter proteins Grb2 and Shc, and EGF-induced activation of the guanine nucleotide exchange factor Sos. The reaction network described by this model contains 356 molecular species and 3749 reactions (see Appendix A, Figure A3 and Supplementary model file for details).

The B-cell receptor (BCR) signaling model has been used to investigate the early events in B-cell antigen receptor signaling [48], particularly the roles of the Src family protein tyrosine kinases Lyn and Fyn, which regulate the activities and fates of B cells. The model includes 1152 species and 24,388 reactions (see Appendix A, Figure A4 and Supplementary model file for details).

Finally, the high-affinity human IgE receptor (Fc*ε*RI) signaling model represents the early events in Fc epsilon receptor (Fc*ε*RI) signaling [49]. This model consists of the interactions between Fc*ε*RI, Lyn, Syk, and a bivalent ligand that aggregates Fc*ε*RI. Several variants of this model have been used previously for testing the performance of some network-free simulators [19]. In this study, we used the variant with 2 *γ* sites in the receptor, which consists of 24 rules, generating a network with 3744 species and 58,276 reactions (see Appendix A, Figure A5).

To include even larger networks, we attempted to use a model of ErbB-mediated activation of the protein kinases ERK and AKT [50] and a model of early T-cell receptor signaling [51]. Both these models are composed of hundreds of rules each, and we were unable to generate the network with BioNetGen because of the excessive memory requirement by this application (in a computer with 32 GB of RAM). Therefore, these were not used for the profiling, and they are examples of systems that currently can only be simulated with network-free methods.

## 5. Results

We performed two sets of tests to probe the performance and scaling of each simulator. The first set was intended to test how the simulators behave in the presence of increasing numbers of particles in the system. This was achieved by setting increasing values for the initial conditions of each species (summarized in Appendix B, Table A1). In the second set, we tested how the simulators scale in increasingly longer simulations, which was achieved by requesting longer end times (Appendix B, Table A1).

### 5.1. Increasing Numbers of Particles

**Figure 1:**
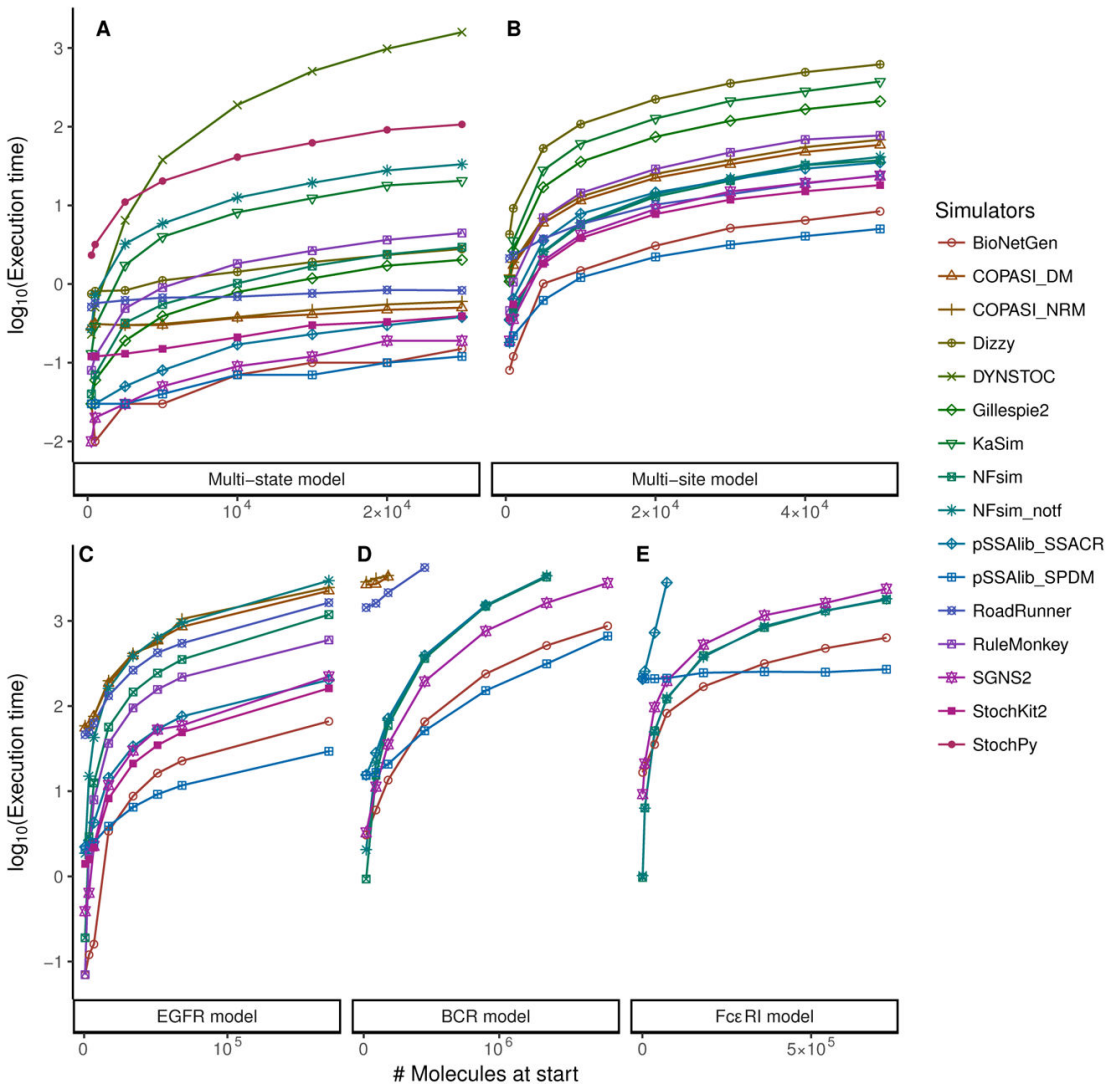
Execution times of the simulators for different number of molecules in the tested models, namely, (**A**) multi-state model, (**B**) multi-site model, (**C**) epidermal growth factor receptor (EGFR) signaling model, (**D**) B-cell receptor (BCR) signaling model, and (**E**) The high-affinity human IgE receptor (Fc*ε*RI) signaling model. In all the models for this test condition, the simulation end time was set to 100 s.

We ran simulations of all the models in Table 2 for a fixed end time of 100 s (simulation time), using the initial conditions in Appendix B, Table A1. Figure 1 depicts the behavior of each simulator for increasing molecule numbers and for each model.

For the multi-state model (Figure 1A), we found that BioNetGen, pSSAlib, and SGNS2 were the fastest. Interestingly, for initial conditions with very few molecules, the network-free implementations KaSim, NFsim, and RuleMonkey were faster than the remaining network-based SSA implementations; however, they become slower than other tools for a larger number of molecules. The execution times of the network-based COPASI (both in DM and NRM), Dizzy, and RoadRunner applications were mostly invariant with the number of molecules, indicating that these tools have a large overhead at the time of loading the model but otherwise were fast. DYNSTOC was fast for very few molecules but very quickly became the slowest, showing the extreme dependency of this approach on the number of particles in the system. Finally StochPy was the second slowest. This was partly perhaps it is written in an interpreted language (Python), but also because this tool outputs every single reaction event, unlike the other tools that allow arbitrary sampling intervals (here we requested them to produce 1000 intervals along the time course; see Methods).

For the multi-site model, the scaling of the execution times with the number of particles was qualitatively similar for all simulators. Although there were clear differences between their execution times, as shown in Figure 1B, there was no clear separation between the network-free and -based implementations. While NFsim was faster than some of network-based simulators, it was slower than BioNetGen, addedpSSAlib, RoadRunner, SGNS2 and StochKit2. These simulators, along with COPASI and RuleMonkey, completed all the simulations within 100 s of a wall-clock time. On the other hand, for large number of particles, Dizzy, Gillespie2 and KaSim took more than 100 s to complete.

In the EGFR signaling model, only 8 of the 13 simulators could complete all simulations within a threshold of 2000 s. For this model, in the lower extreme of the molecule numbers, NFsim and RuleMonkey were the fastest. However with an increasing number of molecules, BioNetGen and pSSAlib_SPDM became the fastest (Figure 1C). Under these conditions, the execution times of NFsim and RuleMonkey became similar to those of COPASI and RoadRunner.

The BCR signaling model could not be simulated using RuleMonkey because of a “Non-binding bimolecular reaction” error (meaning that it could not deal with the complete BNGL). The conversion of the SBML model to the StochKit2 format could not completed, and therefore it was not benchmarked. Among the remaining six simulators, pSSAlib_SPDM was the fastest for a larger number of molecules. For a low number of molecules, pSSAlib_SPDM was slower than BioNetGen, NFsim and SGNS2. All of these closely followed pSSAlib_SPDM, with a similar scaling of their execution times for a larger number of molecules. On the other hand, COPASI and RoadRunner had a visibly large overhead at the start but scaled less dramatically with the increase in the number of particles. For a larger number of particles, COPASI, NFsim, pSSAlib_SSACR and RoadRunner could not complete the simulations within a threshold of 5000 s (wall-clock time).

Both COPASI and RoadRunner were not able to load the Fc*ε*RI signaling model within 5000 s. Although pSSAlib_SPDM had a significant overhead, it had a constant time scaling of the execution time and was the fastest for a large initial number of molecules (See Appendix A, Figure A6); pSSA_SSACR, on the other hand, scaled almost linearly and could not complete all simulations within 5000 s. For the other three simulators, we observed execution time patterns similar to that of the BCR signaling model. BioNetGen was followed by SGNS2, which was marginally slower than NFsim. Given that for this model, the derivation of the network from BNGL takes a considerable amount of time, the choice of using NFsim appears to be advantageous (see Appendix A, Figure A6).

### 5.2. Dependency on the Simulation End Time

**Figure 2:**
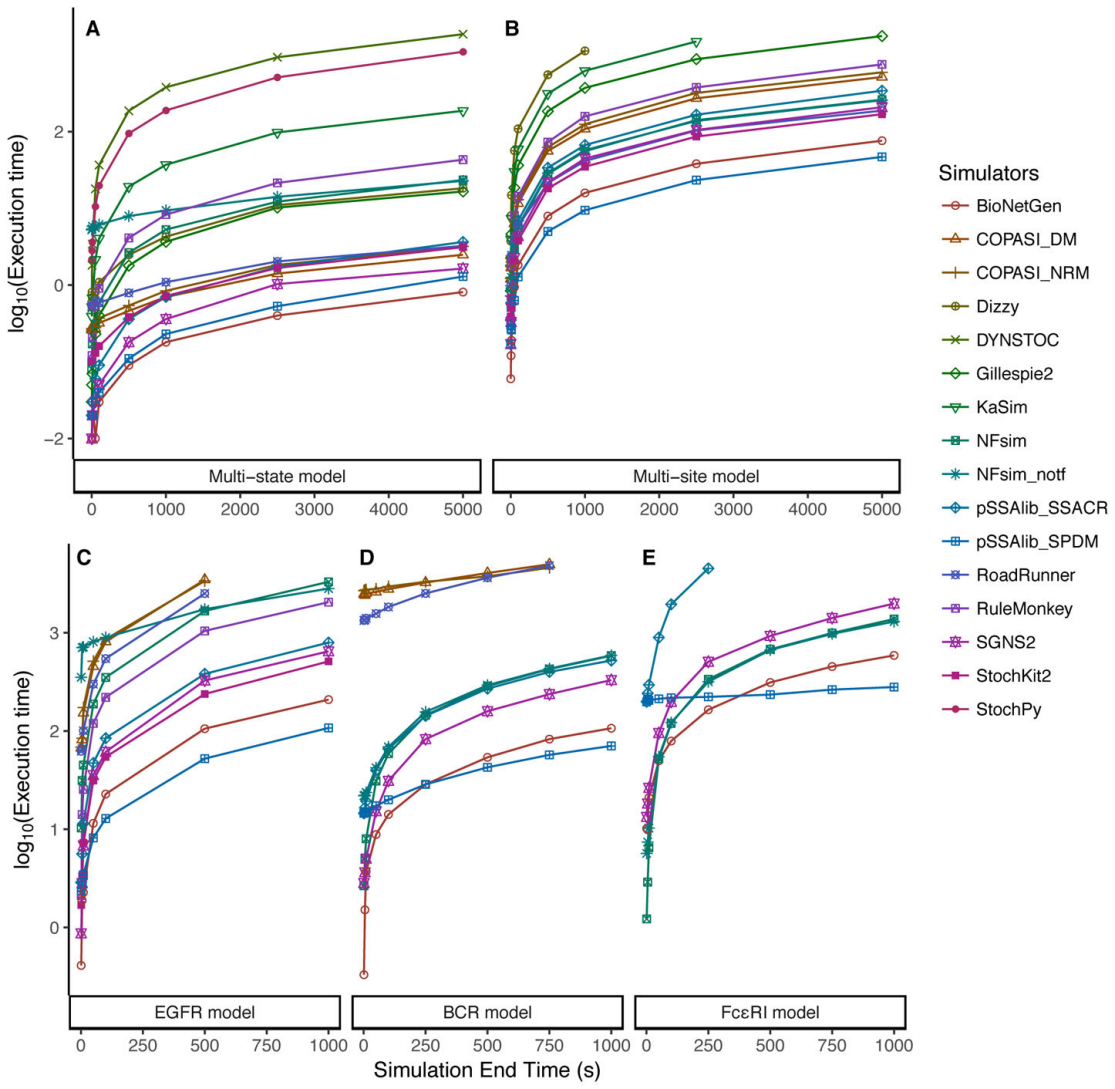
Execution times of the simulators for different simulation end times in the tested models, namely, (**A**) multi-state model, (**B**) multi-site model, (**C**) EGFR signaling model, (**D**) BCR signaling model, and (**E**) Fc*ε*RI signaling model. In all the models for this test condition, the initial number of particles was fixed (see Appendix B, Table A1).

In the second test, we measured the execution times as a function of the simulation end time, keeping the initial number of molecules fixed. Figure 2 depicts the scaling of the execution times with different simulation end times for all the models and simulators.

The results for the multi-state model (Figure 2A) were similar to those of the previous test with increasing initial molecule numbers. Again, DYNSTOC and StochPy were the slowest. For this model, we observed a clear separation of the network-free implementations and the other network-based simulators. More specifically, we found that network-based simulators, except StochPy, were either very fast or invariant with the increasing simulation end time. On the other hand, all the network-free simulators became slower with increasing simulation end time. Also consistent with the previous results on particle numbers, the network-based simulators were the fastest for longer simulation end times.

For the multi-site model (Figure 2B), we found that the execution times of all the simulators scaled similarly to the multi-state model. For this model, only 9 of the 13 simulators completed all simulations within a threshold of 2000 s. DYNSTOC, StochPy, KaSim and Dizzy could not complete all the simulations; pSSAlib_SPDM was the fastest throughout all the simulation end times, followed by BioNetGen, RoadRunner, NFsim, SGNS2 and StochKit2.

For the EGFR signaling model (Figure 2C), COPASI and RoadRunner could not complete all the simulations within 5000 s. Only NFsim and RuleMonkey (network-free) and BioNetGen, pSSAlib, SGNS2 and StochKit2 (network-based) were able to finish all simulations under the time threshold. The network-based simulators were significantly faster than the network-free simulators, whose execution times were in a range similar to those of COPASI and RoadRunner (for the simulations that they could finish).

COPASI and RoadRunner had significantly long execution times for the BCR signaling model (Figure 2D), despite that this increased minimally with the simulation end time. Once again, this is a reflection of these tools’ overhead in model loading and associated “house-keeping tasks”. It should be noted that, as in the first test, RuleMonkey and StochKit could not be used for comparison. Only BioNetGen, NFsim, pSSAlib, and SGNS2 were able to run all the simulations and scale with a similar pattern; pSSAlib_SPDM was the fastest. These simulators were also the only simulators that could run the Fc*ε*RI signaling model in a reasonable time (Figure 2E). Once again, pSSAlib_SPDM had a constant time scaling of the execution time and was the fastest for larger simulation end times; pSSAlib_SSACR could not complete all the simulations within the time threshold of 5000 s. BioNetGen was the second fastest, and only a marginal difference was seen between the execution times of NFsim and SGNS2. In the execution times of the Fc*ε*RI signaling model, it appeared that NFsim was starting to become competitive with the network-based simulators. Given the time taken to generate the network from rules, there could be an advantage to using this network-free simulator for models of this dimension.

## 6. Discussion

Benchmarking several simulators on the basis of different network-based or -free algorithms has revealed interesting patterns. The benchmarks have tested the scaling of the execution times of simulators on the basis of two tests, namely, as a function of the number of particles in the system and as a function of simulation end times. The first test was focused on exposing issues depending on the number of particles in the system; the second test was focused on exposing issues that arised closer to stable states (attractors). We note that most realistic simulations should start from steady states rather than the idealized state of only a few input molecules; biological systems are in stable states at the start of most experiments, which usually apply a perturbation forcing a transition between stable states (either different steady states or stable oscillations). Thus the behaviors over the longer times in the second test were rather important.

The results of both the tests indicate that StochPy and DYNSTOC are the slowest implementations. In the case of StochPy, a SSA–DM implementation, the tool is designed to write the raw simulation output after each event, rather than to do so at a requested fixed-interval output; thus we suspect that it spends a significant amount of time writing (unecessary) output. Additionally, this tool is Python-based, an interpreted language, and this likely also incurres a considerable time penalty. For DYNSTOC, the issue is rather different and is due to the algorithm used, in which each molecule is tracked as an agent. At very low molecule numbers for the simplest model, this tool was among the fastest, and it scaled linearly with the number of molecules. Thus the problem is that this approach is not able to deal with any reasonable number of molecules, which were present in every other model tested. Thus we conclude that such a pure agent-based approach is not competitive.

The results show that, surprisingly, the network-based approach was always the fastest, not only for small- and moderate-sized reaction networks, but also for the larger networks and under all the conditions tested. For the simpler models, with a relatively low number of species and with limited interactions, the lightweight simulators BioNetGen, Gillespie2, pSSAlib_SPDM, and SGNS2 were significantly faster than all the others tested. Gillespie2 became slower when simulating larger models, but BioNetGen, pSSAlib_SPDM, and SGNS2 remained very fast under all conditions tested.

An important aspect of rule-based modeling is the concept of “observables”, which are functions of the species’ abundances. BioNetGen outputs the values of the observables, but SGNS2 only outputs the species abundances; thus in order to obtain values of the observables, the output of SGNS2 would require a further data processing step; pSSAlib outputs data in separate files and requires a post-processing step for this purpose. Although COPASI is slower, mostly as a result of a large overhead in model loading and preparing data structures, it can also readily output the values of the observables. RoadRunner run times are approximately similar to COPASI’s, but it has the same problem of requiring post-processing as in SGNS2.

The network-based simulators tested spanned a range of different SSA approaches. Several were based on the DM (Dizzy, StochPy, Gillespie2, BioNetGen, and COPASI_D), while a couple used the NRM (COPASI_GB and SGNS2) and the CR (pSSAlib_SSACR and StochKit2); pSSAlib_SPDM, which uses PDM, was found to be the fastest for the upper extremes of our tests. In the SPDM, while the time spent on factoring out reaction propensities did not pay off for a small number of molecules or short simulations, it did lead to a significant efficiency when larger numbers of molecules were reached. Consequently, for the Fc*ε*RI signaling model, pSSAlib_SPDM completed the simulation in half the time taken by BioNetGen in the most extreme case tested—a considerable speed-up. The efficient implementation of this method is, however, handicapped by the general usability of the simulator. One setback is that the pSSAlib does not accept any SBML file, but requires specific annotations, which other software do not include. This is particularly problematic for rule-based models that are generated from the BNGL; these models usually have very large numbers of reactions and adding the annotation manually is not practical.

As expected, the DM was observed to be less efficient than the NRM and CR. However, the comparison of the DM against the NRM in the same package (COPASI) showed that the difference is not large and is only apparent under conditions with large numbers of particles. One notable exception to this was BioNetGen, which, while being a DM implementation, was the fastest at all times. This might have been due to its implementation of the sorting variant of the DM. Further efficiency seems to have been obtained by various code optimizations (earlier versions of BioNetGen than that tested here were much slower), but the same is true for SGNS2 (a NRM implementation). We noted, however, that BioNetGen uses the standard C runtime *rand()* function, unlike SGNS2 and most other SSA implementations, which use the Mersenne Twister [52], a much better-quality pseudo-random number generator but that is slower (see Appendix A, Figure A7 for a comparison of the two). The dangers of using poor pseudo-random number generators are well known [53,54], and this could be a concern here, particularly for long simulation times. Also surprising is that the SSA–CR implementations (both pSSAlib_SSACR and SStochKit2) were not faster than the NRM, despite expectations of the contrary [28,30]. The expected advantage of SSA–CR did not materialize in models with large numbers of reactions. Of course, the efficiency of this implementation cannot be ruled out for other types of models not tested here.

What are the advantages of network-free simulation? While the network-free simulators were never the fastest with any of the models and conditions included here, there are clearly situations in which they are needed. The use of network-free simulation is inevitable when the derivation of the network is computationally challenging or impossible. For example, complete models of the ErbB-mediated activation of ERK and AKT [50] and of early T-cell receptor signaling [51] result in very large reaction networks, so large that BioNetGen is unable to generate the corresponding reaction networks. Even if the network could be derived, loading it in simulators such as COPASI and RoadRunner would require a significantly long time. Network-free simulators are also essential to simulate systems with infinitely linking molecules, such as models of polymerization, models of trivalent ligand bivalent receptors (TLBRs), models of large complexes, and so forth. Among the network-free simulators tested, NFsim was generally the fastest. Under some of the less-demanding conditions tested (i.e., low molecule numbers and simpler models), RuleMonkey had a small advantage, but otherwise it is clear that NFsim is currently the best choice. NFsim also provides an option to define functional or conditional rate laws and complex rules. This capability is particularly useful to model systems whose rates are affected by the availability/unavailability of specific molecules. We also identified areas in which network-free simulators require further improvements. For example, we were not able to simulate models with reactions for which several of the bound moieties suffer a transformation (catalysis) with any of the network-free simulators, as these aborted with errors when encountering the catalysis, despite that BioNetGen easily generates their network (as it should). This is a rather common occurrence that happens in every enzyme mechanism (because the reaction between substrates happens with these bound to the enzyme). An example was a model of the electron-transport chain [55], and another was a model of the cap-binding complex in mRNA translation [56]. RuleMonkey was unable to run the BCR signaling model, aborting with the error message “Non-binding bimolecular reaction”. These limitations in processing *valid* BNGL rules affect both NFsim and RuleMonkey, but can hopefully be corrected in future versions of these packages.

The present analysis revealed that the network-based (SPDM) simulation was the most efficient method for all models tested. We hoped to have identified regimes in which network-free simulation would be more competitive. This suggests that, while a rule-based specification of the models is much simpler than enumerating all the reactions, simulation via network-free implementations is not always efficient, unless the derivation of the network from the rules is computationally intractable or there are infinitely linking species/molecules in a model. It is possible that larger models than those tested here (but with a finite number of reactions) may present conditions under which network-free simulation outperforms network-based, but this is yet to be established. On the basis of the present results, we have to conclude that for hybrid algorithms that integrate both of these approaches (e.g., [37]), the only portion of the networks that should be partitioned to be simulated by the network-free approach are those rules leading to the formation of infinite linking chains, while the rest of the network should be simulated using the network-based approach.

## 7. Methods

For each model, we prepared an input file appropriate for each simulator (see below); then we verified that the simulation results quantitatively matched across all simulators (a sample time trace of observables in each model is shown in Appendix A, Figures A1–A5). We found that the numbers of particles output for all the simulators matched. A more thorough test of the exactness of these implementations is beyond this scope, but could be carried out using the SBML stochastic test suite [57].

### 7.1. Model Construction

BNGL specifications for all the models used in this study were retrieved from the respective sources (Table 2). These BNGL files were used as direct input for DYNSTOC, RuleMonkey, and BioNetGen. For NFsim, we generated appropriate XML files from the BNGL with BioNetGen.

Most other simulators used SBML as input; thus we used BioNetGen to generate the network of reactions and write them out in SBML format. We found that the reaction kinetic rate laws in the SBML files generated using BioNetGen were expressed in units of concentration per time, rather than quantity per time as required in the SBML specifications. We created a simple Perl script (see Supplementary file *fixbbionetgensbml.pl*) to correct the BioNetGen SBML Level 2 output. Because a few simulators could only read SBML Level 1, we used COPASI to create translations of the corrected Level 2 BioNetGen file to Level 1 (version 1) versions, which could then be read by RoadRunner, StochPy, Gillespie2, Dizzy and SGNS2.

Two simulators, namely, KaSim and StochKit2, do not accept BNGL nor SBML files as input. For KaSim, the first two model files were directly written in -language and then were used as inputs. Because KaSim was rather slow for these models, we did not create -language files for the remaining three models. For pSSAlib-specific SBML model files, we used a Perl script to fill the required annotations into the SBML model file. StochKit2 has its own format for input, but it is supplied with an SBML translator, *sbmltostochkit*, which was used here to convert the corrected BioNetGen SBML files to its own input format. We note that this converter did not succeed with the BCR and Fc*ε*RI signaling models (failed to finish after a span of 24 h); thus these were not tested with StochKit2.

### 7.2. Simulations

We wanted to test the performance of the simulators with increasing numbers of particles (molecules); thus for each model, we started different runs with increasing initial numbers of molecules (see Appendix B, Table A1). All models were simulated for a fixed end time of 100 s. Another test was to investigate how the simulators behaved with increasing end times, and in this case, the simulations were started with the same fixed number of molecules and were asked to carry out longer simulations each time. In each simulation, the packages were instructed to output 1000 samples at equal intervals along the time course (irrespective of end time). The median values of execution times of five independent runs for each simulator with each model and condition are shown in Figures 1 and 2. In both tests, we observed quantitatively similar trajectories for the observables in the simulation results from all the simulators.

For most of the network-based simulators, Gillespie’s DM was chosen to simulate the models. Both the DM and NRM were used for simulation using COPASI. The NRM method was used for simulation using SGNS2, as it was the only method available in this software. As StochKit2 automatically chooses one method from a wide array of SSA methods on the basis of the input model, to make it comparable with other simulators and to test the constant-time SSA–CR, we adapted the StochKit2 source code to always run the SSA–CR.

The default simulation method was chosen for the network-free simulators. For NFsim, to account for any differences that might have occurred as a result of on-the-fly computation of the observables, we simulated the models in two scenarios: with and without on-the-fly observable computation.

### 7.3. Analysis

The execution times were recorded using *gtime*, a GNU implementation of *time* utility [58]. All simulations were performed in Mac OSX using a 2.9 GHz Intel core i7 processor with 16 GB of RAM.

Data analysis was performed in R [59]. The plots for the scaling of the execution times were generated using the *tidyverse* package [60]. The schematic representations of the models presented in Appendix A, Figures A1–A5 were generated from the respective BNGL files with the software RuleBender [61]. Raw timing data and the analysis scripts can be found in the Supplementary file S1.

## Supplementary Material



## Figures and Tables

**Table 1:** Simulators used in this study. Stochastic simulation algorithm (SSA) used in each of the simulators is listed along with the language they are implemented with.

Approach	Simulator	SSA Method	Language	Version	Reference
Network-based	BioNetGen	SDM *	Perl and C++	2.3.1	[17]
	COPASI_D	DM **	C++	4.21 (Build 166)	[6]
	COPASI_GB	NRM ***	C++	4.21 (Build 166)	[6]
	Dizzy	DM	Java	1.11.4	[9]
	Gillespie2	DM	C	Rev: 56	[10]
	pSSAlib_SPDM	SPDM #	C++	2.0.0	[13]
	pSSAlib_SSACR	CR ##	C++	2.0.0	[13]
	RoadRunner	DM	C	1.4.24	[12]
	SGNS2	NRM	C++	2.1.170	[11]
	StochKit2	CR	C++	2.0.13	[33]
	StochPy	DM	Python	2.3	[8]

Network-free	DYNSTOC	—	C	1.2.0	[25]
	KaSim	—	OCaml	3.5	[22]
	NFsim	—	C++	1.11	[19]
	RuleMonkey	—	C	2.0.25	[39]

* Sorting direct method;

** Direct method;

*** Next reaction method;

# Sorting partial propensity direct method;

## Composition rejection method.

**Table 2:** Models used in this study. The network derivation time with BioNetGen is also shown for each of the models.

Model	No. of Species	No. of Rules	No. of Reactions	Derivation Time (s)
Multi-state [17,25]	6	4	8	0.0
Multi-site [39]	66	12	288	0.3

EGFR * signaling [47]	356	23	3749	11.6
BCR ** signaling [48]	1122	72	24,388	33.17
Fc*ε*RI *** signaling (*γ*) [49]	3744	24	58,276	163.8

* Epidermal growth factor receptor;

** B-cell receptor;

*** The high-affinity human IgE receptor.
