# Supplementary material for: An Overview of Network-Based and -Free Approaches for Stochastic Simulation of Biochemical Systems
Source: Computation (Basel). Author manuscript; Available in PMC 2018 Jun 21. (PMC6013266; doi:10.3390/computation6010009)
Supplement: Supplementary file 1 [file NIHMS975398-supplement-supplement_1.pdf]

The default simulation method was chosen for the network-free simulators. For NFsim, to account for any differences that might have occurred as a result of on-the-fly computation of the observables, we simulated the models in two scenarios: with and without on-the-fly observable computation.

7.3. Analysis

The execution times were recorded using *gtime*, a GNU implementation of *time* utility [58]. All simulations were performed in Mac OSX using a 2.9 GHz Intel core i7 processor with 16 GB of RAM.

Data analysis was performed in R [59]. The plots for the scaling of the execution times were generated using the *tidyverse* package [60]. The schematic representations of the models presented in Appendix A, Figures A1–A5 were generated from the respective BNGL files with the software RuleBender [61]. Raw timing data and the analysis scripts can be found in the Supplementary file S1.

Supplementary Materials: The following are available online at www.mdpi.com/2079-3197/6/1/9/s1: File S1: archive containing model files, raw timing data, and analysis scripts.

Acknowledgments: We are grateful to the National Institutes of Health (NIGMS) for funding this work under Grant No. GM080219. We thank Carole Proctor and Colin Gillespie for supplying the source code of the Gillespie2 software.

Author Contributions: A.G. and P.M. conceived and designed the experiments and wrote the paper; A.G. performed the experiments and analyzed the data.

Conflicts of Interest: A.G. is one of the authors of SGNS2; P.M. is one of the authors of COPASI. The authors declare no financial conflict of interest. The founding sponsors had no role in the design of the study; in the collection, analyses, or interpretation of data; in the writing of the manuscript; or in the decision to publish the results.

Appendix A. Supplementary Figures

Appendix A.1. Multi-State Model

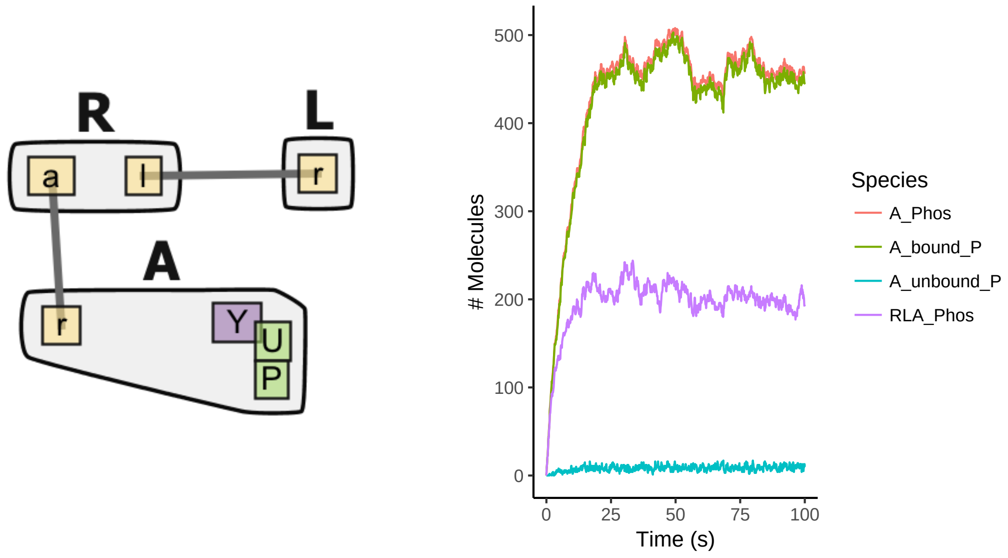

Figure A1. Multi-state model and its simulation. (**Left**) Schematic illustration of multi-state model, in which species R binds with L to form an R.L composite. The R.L composite, at a different rate, unbinds to result in R and L. An additional species A can bind with R of the binding model and has a phosphorylation site. Figure generated using RuleBender. (**Right**) An example time trace of the observables of this model.

Appendix A.2. Multi-Site Model

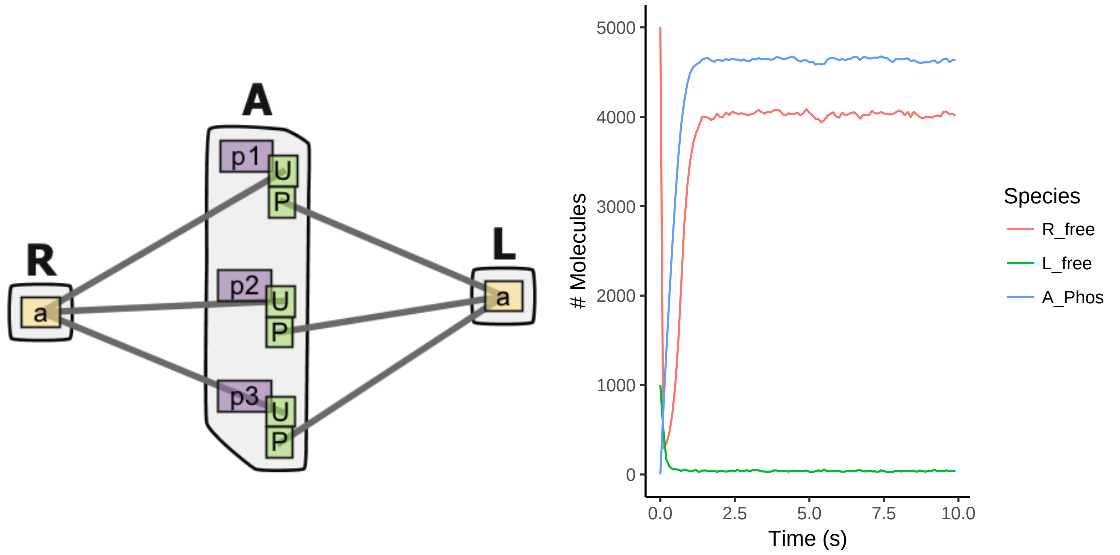

Figure A2. Multi-site model and its simulation. (**Left**) Schematic illustration of multi-site model. In the multi-state model, species A has two additional phosphorylation sites, where both R and L can bind. Figure generated using RuleBender. (**Right**) An example time trace of the observables of this model.

Appendix A.3. EGFR Signaling Model

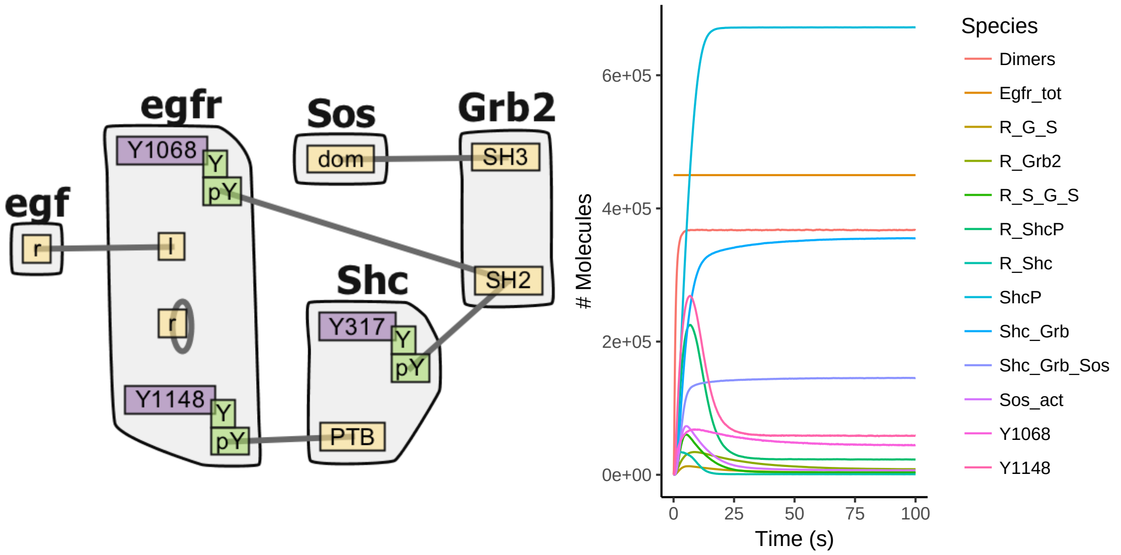

Figure A3. Epidermal growth factor receptor (EGFR) signaling model and its simulation. (**Left**) Schematic illustration of the EGFR signaling model. Figure generated using RuleBender. (**Right**) An example time trace of the observables of this model.

Appendix A.4. BCR Signaling Model

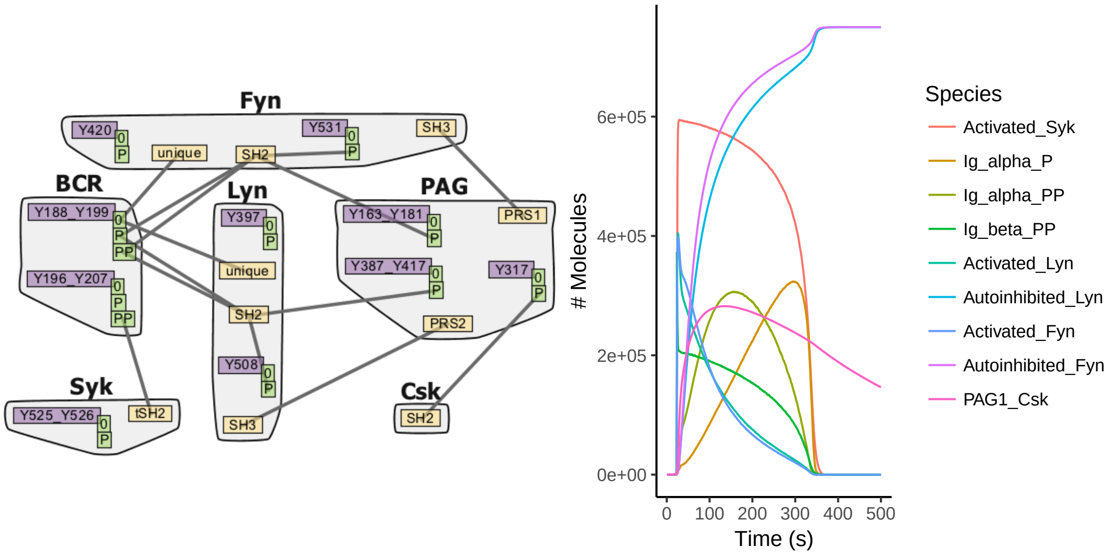

Figure A4. B-cell receptor (BCR) signaling model and its simulation. (**Left**) Schematic illustration of the BCR signaling model. Figure generated using RuleBender. (**Right**) An example time trace of the observables of this model.

Appendix A.5. FcϵRI Signaling Model

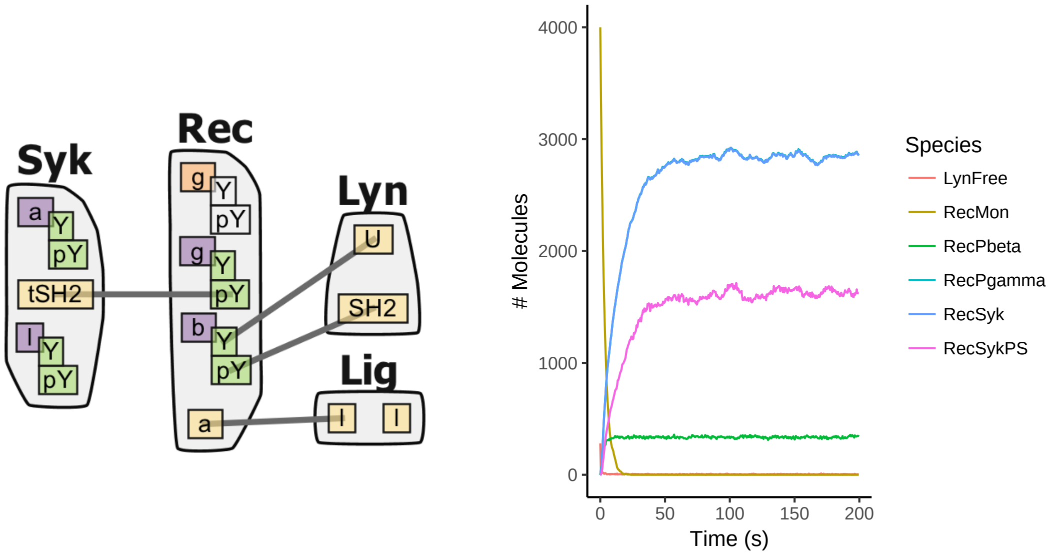

Figure A5. FcϵRI signaling model and its simulation. (**Left**) Schematic illustration of the FcϵRI signaling model. Figure generated using RuleBender. (**Right**) An example time trace of the observables of this model.

Appendix A.6. Fastest Simulators under the Tested Scenarios

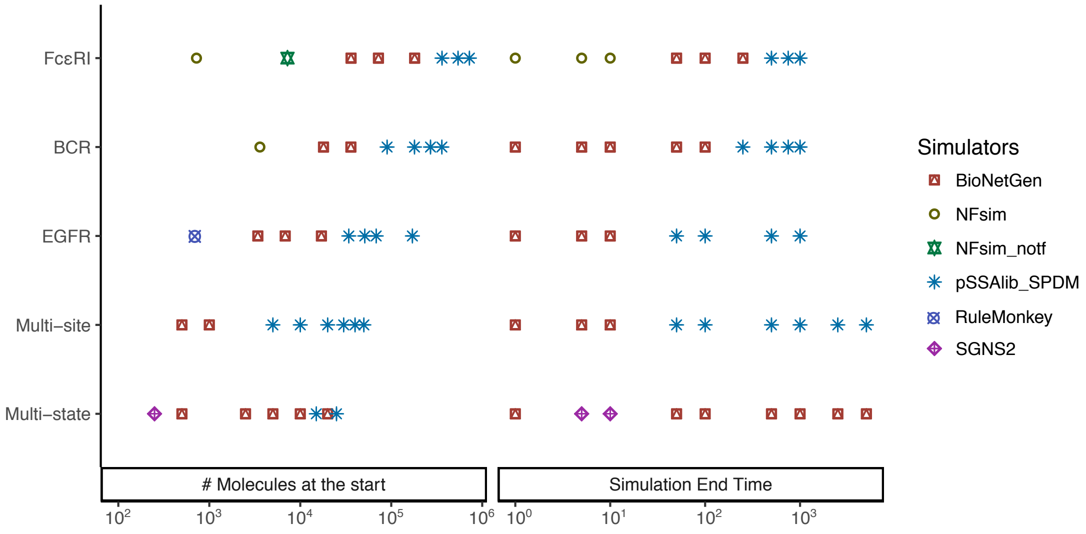

Figure A6. For each of the tested conditions of all five models, the simulator that took the least amount of time is shown for both (**Left**) different molecule numbers and (**Right**) different simulation end times.

Appendix A.7. Performance Differences between Random Number Generators

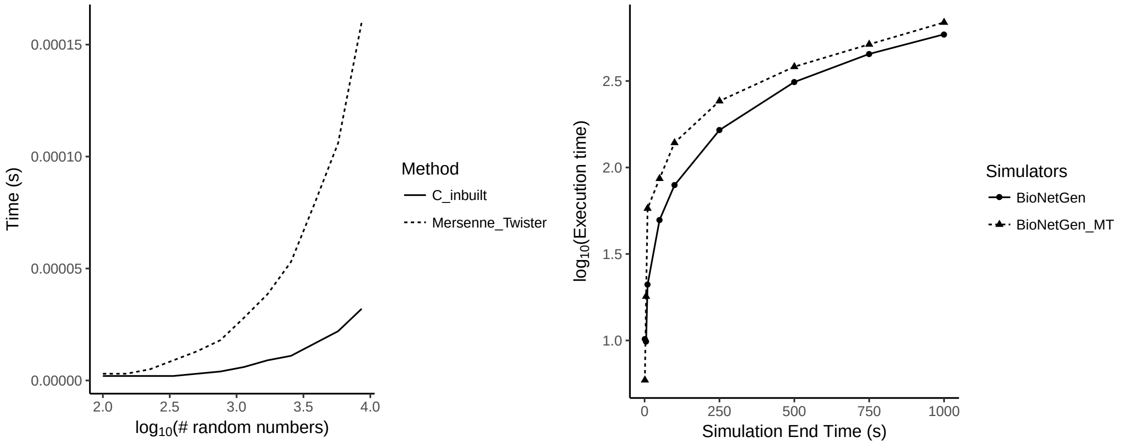

Figure A7. Effects due to selection of different random number generators. (**Left**) Comparison of pseudo-random number generation times for the C runtime *rand()* function (solid line) and the Mersenne Twister (dashed line). (**Right**) Difference in performance of BioNetGen using the C runtime *rand()* function (solid line) and a modified version using the Mersenne Twister (dashed line).

Appendix B. Supplementary Table

Table A1. Test scenarios for different models.

Model	Test Scenario	Number of Molecules	Simulation End Time (s)
Multi-state	Different molecule numbers	R = 500 to 25,000<br>L = 100 to 10,000<br>A = 500 to 25,000	100
	Different simulation end times	R = 5000, L = 1000, A = 5000	1 to 10,000
Multi-site	Different molecule numbers	R = 500 to 25,000<br>L = 100 to 10,000<br>A = 500 to 25,000	100
	Different simulation end times	R = 5000, L = 1000, A = 5000	1 to 10,000
EGFR	Different molecule numbers	egf_{tot} = 1.2 × 10^4 to 6.0 × 10^6<br>$egfr_{tot}$ = 1800 to 9.0 × 10^5<br>$Grb2_{tot}$ = 1000 to 5.0 × 10^5<br>Shc_{tot} = 2700 to 1.35 × 10^6<br>Sos_{tot} = 130 to 6.5 × 10^4<br>$Grb2\_Sos_{tot}$ = 490 to 2.45 × 10^4	100
	Different simulation end times	egf_{tot}= 1.2 × 10^5<br>$egfr_{tot}$ = 1.8 × 10^5<br>$Grb2_{tot}$ = 1.0 × 10^5<br>Shc_{tot} = 2.7 × 10^5<br>Sos_{tot} = 1.3 × 10^4<br>$Grb2\_Sos_{tot}$ = 4.9 × 10^4	1 to 1000
BCR	Different molecule numbers	$p1$ = 3000 to 7.5 × 10^5	100
	Different simulation end times	$p1$ = 30,000	1 to 1000
FcϵRI	Different molecule numbers	Lig_{tot} = 6000 to 600,000<br>Rec_{tot} = 400 to 40,000<br>Lyn_{tot} = 30 to 3000<br>Syk_{tot} = 400 to 40,000	100
	Different simulation end times	Lig_{tot} = 60,000<br>Rec_{tot} = 4000<br>Lyn_{tot} = 300<br>Syk_{tot} = 4000	1 to 1000

References

1. McQuarrie, D. Stochastic approach to chemical kinetics. *J. Appl. Probab.* **1967**, *4*, 413–478.
2. Gillespie, D.T. A general method for numerically simulating coupled chemical reactions. *J. Comput. Phys.* **1976**, *22*, 403–434.
3. Gillespie, D.T. Exact Stochastic Simulation of Coupled Chemical Reactions. *J. Phys. Chem.* **1977**, *81*, 2340–2361.
4. Gibson, M.A.; Bruck, J. Efficient Exact Stochastic Simulation of Chemical Systems with Many Species and Many Channels. *J. Phys. Chem.* **2000**, *104*, 1876–1889.
5. Cao, Y.; Li, H.; Petzold, L.; Bruck, J. Efficient formulation of the stochastic simulation algorithm for chemically reacting systems. *J. Chem. Phys.* **2004**, *121*, 4059–4067.
6. Hoops, S.; Sahle, S.; Gauges, R.; Lee, C.; Pahle, J.; Simus, N.; Singhal, M.; Xu, L.; Mendes, P.; Kummer, U. COPASI—A COmplex PAthway SImulator. *Bioinformatics* **2006**, *22*, 3067–3074.
7. Blinov, M.L.; Schaff, J.C.; Vasilescu, D.; Moraru, I.I.; Bloom, J.E.; Loew, L.M. Compartmental and Spatial Rule-Based Modeling with Virtual Cell. *Biophys. J.* **2017**, *113*, 1365–1372.
8. Maarleveld, T.R.; Olivier, B.G.; Bruggeman, F.J. StochPy: A Comprehensive, User-Friendly Tool for Simulating Stochastic Biological Processes. *PLoS ONE* **2013**, *8*, e79345.

9. Ramsey, S.; Orrell, D.; Bolouri, H. Dizzy: Stochastic simulation of large-scale genetic regulatory networks. *J. Bioinform. Comput. Biol.* **2005**, *3*, 415–436.
10. Gillespie, C.S.; Wilkinson, D.J.; Proctor, C.J.; Shanley, D.P.; Boys, R.J.; Kirkwood, T.B.L. Tools for the SBML Community. *Bioinformatics* **2006**, *22*, 628–629.
11. Lloyd-Price, J.; Gupta, A.; Ribeiro, A.S. SGNS2: A compartmentalized stochastic chemical kinetics simulator for dynamic cell populations. *Bioinformatics* **2012**, *28*, 3004–3005.
12. Somogyi, E.T.; Bouteiller, J.M.; Glazier, J.A.; König, M.; Medley, J.K.; Swat, M.H.; Sauro, H.M. libRoadRunner: A high performance SBML simulation and analysis library. *Bioinformatics* **2015**, *31*, 3315–3321.
13. Ostrenko, O.; Incardona, P.; Ramaswamy, R.; Brusch, L.; Sbalzarini, I.F. pSSAlib: The partial-propensity stochastic chemical network simulator. *PLoS Comput. Biol.* **2017**, *13*, e1005865.
14. Hlavacek, W.S.; Faeder, J.R.; Blinov, M.L.; Perelson, A.S.; Goldstein, B. The complexity of complexes in signal transduction. *Biotechnol. Bioeng.* **2003**, *84*, 783–794.
15. Hlavacek, W.S.; Faeder, J.R.; Blinov, M.L.; Posner, R.G.; Hucka, M.; Fontana, W. Rules for Modeling Signal-Transduction Systems. *Sci. Signal.* **2006**, *2006*, doi:10.1126/stke.3442006re6.
16. Stefan, M.I.; Bartol, T.M.; Sejnowski, T.J.; Kennedy, M.B. Multi-state Modeling of Biomolecules. *PLoS Comput. Biol.* **2014**, *10*, e1003844.
17. Blinov, M.L.; Faeder, J.R.; Goldstein, B.; Hlavacek, W.S. BioNetGen: Software for rule-based modeling of signal transduction based on the interactions of molecular domains. *Bioinformatics* **2004**, *20*, 3289–3291.
18. Danos, V.; Laneve, C. Formal molecular biology. *Theor. Comput. Sci.* **2004**, *325*, 69–110.
19. Sneddon, M.W.; Faeder, J.R.; Emonet, T. Efficient modeling, simulation and coarse-graining of biological complexity with NFsim. *Nat. Methods* **2010**, *8*, 177–183.
20. Chylek, L.A.; Harris, L.A.; Tung, C.S.; Faeder, J.R.; Lopez, C.F.; Hlavacek, W.S. Rule-based modeling: A computational approach for studying biomolecular site dynamics in cell signaling systems. *Wiley Interdiscip. Rev. Syst. Biol. Med.* **2014**, *6*, 13–36.
21. Chylek, L.A.; Harris, L.A.; Faeder, J.R.; Hlavacek, W.S. Modeling for (physical) biologists: An introduction to the rule-based approach. *Phys. Biol.* **2015**, *12*, 045007.
22. Danos, V.; Feret, J.; Fontana, W.; Krivine, J. Scalable Simulation of Cellular Signaling Networks. *Lect. Notes Comput. Sci.* **2007**, *4807*, 139–157.
23. Harris, L.A.; Hogg, J.S.; Tapia, J.J.; Sekar, J.A.P.; Gupta, S.; Korsunsky, I.; Arora, A.; Barua, D.; Sheehan, R.P.; Faeder, J.R. BioNetGen 2.2: Advances in rule-based modeling. *Bioinformatics* **2016**, *32*, 3366–3368.
24. Lopez, C.F.; Muhlich, J.L.; Bachman, J.A.; Sorger, P.K. Programming biological models in Python using PySB. *Mol. Syst. Biol.* **2013**, *9*, doi:10.1038/msb.2013.1.
25. Colvin, J.; Monine, M.I.; Faeder, J.R.; Hlavacek, W.S.; Von Hoff, D.D.; Posner, R.G. Simulation of large-scale rule-based models. *Bioinformatics* **2009**, *25*, 910–917.
26. McCollum, J.M.; Peterson, G.D.; Cox, C.D.; Simpson, M.L.; Samatova, N.F. The sorting direct method for stochastic simulation of biochemical systems with varying reaction execution behavior. *Comput. Biol. Chem.* **2006**, *30*, 39–49.
27. Ramaswamy, R.; González-Segredo, N.; Sbalzarini, I.F. A new class of highly efficient exact stochastic simulation algorithms for chemical reaction networks. *J. Chem. Phys.* **2009**, *130*, 244104.
28. Slepoy, A.; Thompson, A.P.; Plimpton, S.J. A constant-time kinetic Monte Carlo algorithm for simulation of large biochemical reaction networks. *J. Chem. Phys.* **2008**, *128*, 205101.
29. Ramaswamy, R.; Sbalzarini, I.F. A partial-propensity variant of the composition-rejection stochastic simulation algorithm for chemical reaction networks. *J. Chem. Phys.* **2010**, *132*, 044102.
30. Thanh, V.H.; Zunino, R.; Priami, C. On the rejection-based algorithm for simulation and analysis of large-scale reaction networks. *J. Chem. Phys.* **2015**, *142*, 244106.
31. Gillespie, D.T. Approximate accelerated stochastic simulation of chemically reacting systems. *J. Chem. Phys.* **2001**, *115*, 1716–1733.
32. Pahle, J. Biochemical simulations: stochastic, approximate and hybrid approaches. *Brief. Bioinform.* **2009**, *10*, 53–64.
33. Sanft, K.R.; Wu, S.; Roh, M.; Fu, J.; Lim, R.K.; Petzold, L.R. StochKit2: Software for discrete stochastic simulation of biochemical systems with events. *Bioinformatics* **2011**, *27*, 2457–2458.
34. Palmisano, A.; Hoops, S.; Watson, L.T.; Jones, T.C., Jr.; Tyson, J.J.; Shaffer, C.A. Multistate Model Builder (MSMB): A flexible editor for compact biochemical models. *BMC Syst. Biol.* **2014**, *8*, doi:10.1186/ 1752-0509-8-42.

35. Faeder, J.R.; Blinov, M.L.; Goldstein, B.; Hlavacek, W.S. Rule-based modeling of biochemical networks. *Complexity* **2005**, *10*, 22–41.
36. Blinov, M.L.; Yang, J.; Faeder, J.R.; Hlavacek, W.S. Graph Theory for Rule-Based Modeling of Biochemical Networks. *Trans. Comput. Syst. Biol.* **2006**, *7*, 89–106.
37. Hogg, J.S.; Harris, L.A.; Stover, L.J.; Nair, N.S.; Faeder, J.R. Exact Hybrid Particle/Population Simulation of Rule-Based Models of Biochemical Systems. *PLoS Comput. Biol.* **2014**, *10*, e1003544.
38. Andrei, O.; Kirchner, H. A Rewriting Calculus for Multigraphs with Ports. *Electron. Notes Theor. Comput. Sci.* **2008**, *219*, 67–82.
39. Colvin, J.; Monine, I.M.; Gutenkunst, R.N.; Hlavacek, W.S.; Hoff, D.D.V.; Posner, R.G. RuleMonkey: Software for stochastic simulation of rule-based models. *BMC Bioinform.* **2010**, *11*, 404.
40. Novere, N.L.; Shimizu, T.S. STOCHSIM: Modelling of stochastic biomolecular processes. *Bioinformatics* **2001**, *17*, 575–576.
41. Yang, J.; Hlavacek, W.S. Efficiency of reactant site sampling in network-free simulation of rule-based models for biochemical systems. *Phys. Biol.* **2011**, *8*, doi:10.1088/1478-3975/8/5/055009.
42. Yang, J.; Monine, M.I.; Faeder, J.R.; Hlavacek, W.S. Kinetic Monte Carlo method for rule-based modeling of biochemical networks. *Phys. Rev. E* **2008**, *78*, 031910.
43. Falkenberg, C.V.; Blinov, M.L.; Loew, L.M. Pleomorphic Ensembles: Formation of Large Clusters Composed of Weakly Interacting Multivalent Molecules. *Biophys. J.* **2013**, *105*, 2451–2460.
44. Lok, L.; Brent, R. Automatic generation of cellular reaction networks with Moleculizer 1.0. *Nat. Biotechnol.* **2005**, *23*, 131–136.
45. Blinov, M.L.; Faeder, J.R.; Yang, J.; Goldstein, B.; Hlavacek, W.S. 'On-the-fly' or 'generate-first' modeling? *Nat. Biotechnol.* **2005**, *23*, 1344–1345.
46. Hucka, M.; Finney, A.; Sauro, H.M.; Bolouri, H.; Doyle, J.C.; Kitano, H.; Arkin, A.P.; Bornstein, B.J.; Bray, D.; Cornish-Bowden, A.; et al. The systems biology markup language (SBML): A medium for representation and exchange of biochemical network models. *Bioinformatics* **2003**, *19*, 524–531.
47. Blinov, M.L.; Faeder, J.R.; Goldstein, B.; Hlavacek, W.S. A network model of early events in epidermal growth factor receptor signaling that accounts for combinatorial complexity. *Biosystems* **2006**, *83*, 136–151.
48. Barua, D.; Hlavacek, W.S.; Lipniacki, T. A Computational Model for Early Events in B Cell Antigen Receptor Signaling: Analysis of the Roles of Lyn and Fyn. *J. Immunol.* **2012**, *189*, 646–658.
49. Faeder, J.R.; Hlavacek, W.S.; Reischl, I.; Blinov, M.L.; Metzger, H.; Redondo, A.; Wofsy, C.; Goldstein, B. Investigation of Early Events in FcϵRI-Mediated Signaling Using a Detailed Mathematical Model. *J. Immunol.* **2003**, *170*, 3769–3781.
50. Creamer, M.S.; Stites, E.C.; Aziz, M.; Cahill, J.A.; Tan, C.W.; Berens, M.E.; Han, H.; Bussey, K.J.; Von Hoff, D.D.; Hlavacek, W.S.; et al. Specification, annotation, visualization and simulation of a large rule-based model for ERBB receptor signaling. *BMC Syst. Biol.* **2012**, *6*, doi:10.1186/1752-0509-6-107.
51. Chylek, L.A.; Akimov, V.; Dengjel, J.; Rigbolt, K.T.G.; Hu, B.; Hlavacek, W.S.; Blagoev, B. Phosphorylation Site Dynamics of Early T-cell Receptor Signaling. *PLoS ONE* **2014**, *9*, e104240.
52. Matsumoto, M.; Nishimura, T. Mersenne Twister: A 623-Dimensionally Equidistributed Uniform Pseudo-Random Number Generator. *ACM Trans. Model. Comput. Simul.* **1998**, *8*, 3–30.
53. Marsaglia, G. Random Numbers Fall Mostly in the Planes. *Proc. Natl. Acad. Sci. USA* **1968**, *61*, 25–28.
54. Park, S.K.; Miller, K.W. Random Numbers Generators: Good Ones Are Hard To Find. *Commun. Assoc. Comput. Mach.* **1988**, *31*, 1192–1201.
55. Selivanov, V.A.; Votyakova, T.V.; Zeak, J.A.; Trucco, M.; Roca, J.; Cascante, M. Bistability of Mitochondrial Respiration Underlies Paradoxical Reactive Oxygen Species Generation Induced by Anoxia. *PLoS Comput. Biol.* **2009**, *5*, e1000619.
56. Meng, X.; Firczuk, H.; Pietroni, P.; Westbrook, R.; Dacheux, E.; Mendes, P.; McCarthy, J.E. Minimum-noise production of translation factor eIF4G maps to a mechanistically determined optimal rate control window for protein synthesis. *Nucleic Acids Res.* **2017**, *45*, 1015–1025.
57. Evans, T.W.; Gillespie, C.S.; Wilkinson, D.J. The SBML discrete stochastic models test suite. *Bioinformatics* **2008**, *24*, 285–286.
58. GNU Implementation of Time. Available online: https://www.gnu.org/software/time/ (accessed on 22 October 2017).

59. R Core Team. *R: A Language and Environment for Statistical Computing*; R Foundation for Statistical Computing: Vienna, Austria, 2017.
60. Wickham, H. *Tidyverse: Easily Install and Load the 'Tidyverse'*, version 1.2.1; R Package. Available online: https://www.tidyverse.org/ (accessed on 15 November 2017).
61. Xu, W.; Smith, A.M.; Faeder, J.R.; Marai, G.E. RuleBender: A visual interface for rule-based modeling. *Bioinformatics* **2011**, *27*, 1721–1722.

© 2018 by the authors. Licensee MDPI, Basel, Switzerland. This article is an open access article distributed under the terms and conditions of the Creative Commons Attribution (CC BY) license (http://creativecommons.org/licenses/by/4.0/).
